# *N*-(2,3-Dimethyl­phen­yl)-2,2,2-trimethyl­acetamide

**DOI:** 10.1107/S1600536809021229

**Published:** 2009-06-10

**Authors:** B. Thimme Gowda, Sabine Foro, Hiromitsu Terao, Hartmut Fuess

**Affiliations:** a Department of Chemistry, Mangalore University, Mangalagangotri 574 199, Mangalore, India; b Institute of Materials Science, Darmstadt University of Technology, Petersenstrasse 23, D-64287 Darmstadt, Germany; c Faculty of Integrated Arts and Sciences, Tokushima University, Minamijosanjima-cho, Tokushima 770-8502, Japan

## Abstract

The N—H bond in the title compound, C_13_H_19_NO, is *anti* to the C=O bond and is also *anti* to both the 2- and 3-methyl substituents in the aromatic ring. In the crystal, inter­molecular N—H⋯O hydrogen bonds link the mol­ecules into chains propagating along the *c* axis.

## Related literature

For the preparation of the title compound, see: Shilpa & Gowda (2007 ▶). For related structures, see: Gowda *et al.* (2007**a* ▶,*b* ▶,c* ▶).
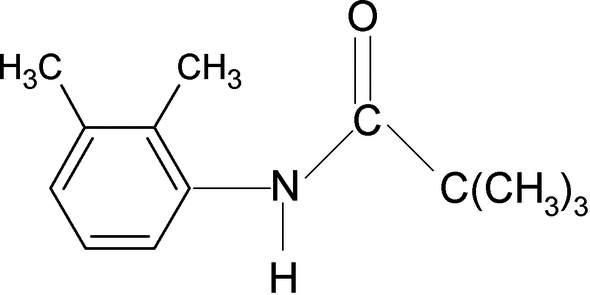


## Experimental

### 

#### Crystal data

C_13_H_19_NO
*M* *_r_* = 205.29
Monoclinic, 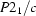

*a* = 18.276 (4) Å
*b* = 8.227 (2) Å
*c* = 8.633 (2) Å
β = 97.94 (2)°
*V* = 1285.6 (5) Å^3^
*Z* = 4
Mo *K*α radiation
μ = 0.07 mm^−1^
*T* = 299 K
0.45 × 0.16 × 0.08 mm

#### Data collection

Oxford Diffraction Xcalibur diffractometer with a Sapphire CCD detector
Absorption correction: multi-scan (*CrysAlis RED*; Oxford Diffraction, 2007 ▶) *T*_min_ = 0.971, *T*_max_ = 0.992
4295 measured reflections
2349 independent reflections
1214 reflections with *I* > 2σ(*I*)
*R*_int_ = 0.047

#### Refinement

*R*[*F* ^2^ > 2σ(*F* ^2^)] = 0.073
*wR*(*F* ^2^) = 0.221
*S* = 0.96
2349 reflections
195 parameters
112 restraints
H-atom parameters constrained
Δρ_max_ = 0.28 e Å^−3^
Δρ_min_ = −0.24 e Å^−3^

### 

Data collection: *CrysAlis CCD* (Oxford Diffraction, 2004 ▶); cell refinement: *CrysAlis RED* (Oxford Diffraction, 2007 ▶); data reduction: *CrysAlis RED*; program(s) used to solve structure: *SHELXS97* (Sheldrick, 2008 ▶); program(s) used to refine structure: *SHELXL97* (Sheldrick, 2008 ▶); molecular graphics: *ORTEP-3* for Windows (Farrugia, 1997 ▶) and *PLATON* (Spek, 2009 ▶); software used to prepare material for publication: *SHELXL97*.

## Supplementary Material

Crystal structure: contains datablocks I, global. DOI: 10.1107/S1600536809021229/ci2806sup1.cif

Structure factors: contains datablocks I. DOI: 10.1107/S1600536809021229/ci2806Isup2.hkl

Additional supplementary materials:  crystallographic information; 3D view; checkCIF report

## Figures and Tables

**Table 1:** Hydrogen-bond geometry (Å, °)

*D*—H⋯*A*	*D*—H	H⋯*A*	*D*⋯*A*	*D*—H⋯*A*
N1—H1*N*⋯O1^i^	0.94	2.11	2.966 (3)	151

Symmetry code: (i) 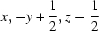.

**Fig. 1.**
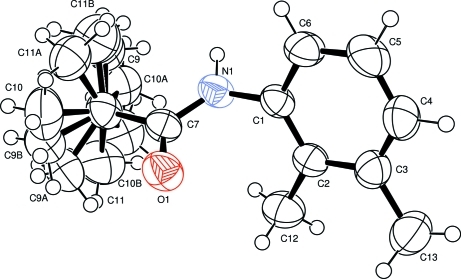
Molecular structure of the title compound, showing the atom labelling scheme. Displacement ellipsoids are drawn at the 50% probability level. All disorder components are shown.

**Fig. 2.**
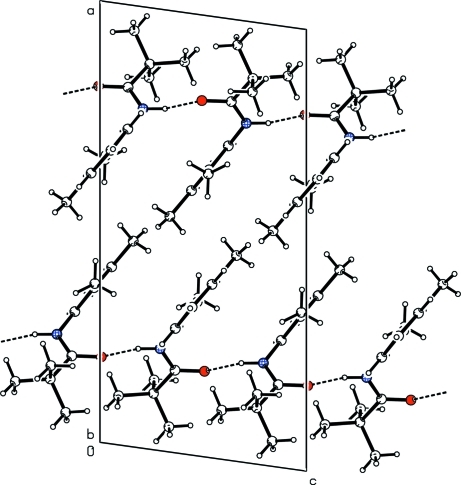
Molecular packing of the title compound, viewed down the b axis. Only the major disorder component is shown. Hydrogen bonds are shown as dashed lines.

## supplementary crystallographic information

### Comment

As part of a study of the effect of ring and side chain substitutions on the crystal structures of chemically and biologically important class of compounds such as aromatic amides (Gowda *et al.*, 2007*a, b, c*), the crystal structure of 2,2,2-trimethyl-*N*-(2,3-dimethylphenyl)-acetamide has been determined.

The conformation of the N–H bond in the title compound is *anti* to both the 2- and 3-methyl substituents in the aromatic ring (Fig. 1), in contrast to the *syn* conformation observed with respect to both the 2- and 3-chloro substituents in 2,2,2-trimethyl-*N*-(2,3-dichlorophenyl)acetamide (Gowda *et al.*, 2007*a*), *syn* conformation with respect to the 2-methyl substituent in 2,2,2-trimethyl-*N*- (2-methylphenyl)acetamide (Gowda *et al.*, 2007*b*) and *anti* conformation with respect to 3-methyl substituent in 2,2,2-trimethyl-*N*- (3-methylphenyl)acetamide (Gowda *et al.*, 2007*c*). Furthermore, the conformation of the C═O bond is *anti* to the N—H bond in the amide segment.

In the title compound, the molecules are linked into chains (Fig. 2) running along the *c* axis by intermolecular N—H···O hydrogen bonds (Table 1).

### Experimental

The title compound was prepared according to the literature method (Shilpa & Gowda, 2007). The purity of the compound was checked by determining its melting point. It was characterized by recording its infrared and NMR spectra (Shilpa & Gowda, 2007). Single crystals of the title compound were grown by slow evaporation of its ethanolic solution at room temperature.

### Refinement

The tert-butyl group is disordered over three orientations with occupancies of 0.743 (14), 0.153 (7) and 0.104 (13). All C—C/C···C distances involving disordered atoms were restrained to be equal and also they were subjected to a rigid bond restraint. The U^ij^ components of the disordered atoms were restrained to approximate isotropic behaviour. The N-bound H atom was located in a difference map and was allowed to ride on the N atom. The remaining H atoms were positioned geometrically and refined using a riding model [C-H = 0.93–0.96 Å]. The U_iso_ parameter for all H atoms were set to 1.2 times of the *U*_eq_ of the parent atom.

### Crystal data

**Table:**

C_13_H_19_NO	*F*(000) = 448
*M**_r_* = 205.29	*D*_x_ = 1.061 Mg m^−^^3^
Monoclinic, *P*2_1_/*c*	Mo *K*α radiation, λ = 0.71073 Å
Hall symbol: -P 2ybc	Cell parameters from 1033 reflections
*a* = 18.276 (4) Å	θ = 2.7–27.9°
*b* = 8.227 (2) Å	µ = 0.07 mm^−^^1^
*c* = 8.633 (2) Å	*T* = 299 K
β = 97.94 (2)°	Needle, colourless
*V* = 1285.6 (5) Å^3^	0.45 × 0.16 × 0.08 mm
*Z* = 4	

### Data collection

**Table:**

Oxford Diffraction Xcalibur diffractometer with a Sapphire CCD detector	2349 independent reflections
Radiation source: fine-focus sealed tube	1214 reflections with *I* > 2σ(*I*)
graphite	*R*_int_ = 0.047
Rotation method data acquisition using ω and φ scans	θ_max_ = 25.4°, θ_min_ = 2.7°
Absorption correction: multi-scan (*CrysAlis RED*; Oxford Diffraction, 2007)	*h* = −17→21
*T*_min_ = 0.971, *T*_max_ = 0.992	*k* = −6→9
4295 measured reflections	*l* = −10→8

### Refinement

**Table:**

Refinement on *F*^2^	Primary atom site location: structure-invariant direct methods
Least-squares matrix: full	Secondary atom site location: difference Fourier map
*R*[*F*^2^ > 2σ(*F*^2^)] = 0.073	Hydrogen site location: inferred from neighbouring sites
*wR*(*F*^2^) = 0.221	H-atom parameters constrained
*S* = 0.96	*w* = 1/[σ^2^(*F*_o_^2^) + (0.1311*P*)^2^] where *P* = (*F*_o_^2^ + 2*F*_c_^2^)/3
2349 reflections	(Δ/σ)_max_ = 0.001
195 parameters	Δρ_max_ = 0.28 e Å^−^^3^
112 restraints	Δρ_min_ = −0.24 e Å^−^^3^

### Special details

**Table:**

Geometry. All e.s.d.'s (except the e.s.d. in the dihedral angle between two l.s. planes) are estimated using the full covariance matrix. The cell e.s.d.'s are taken into account individually in the estimation of e.s.d.'s in distances, angles and torsion angles; correlations between e.s.d.'s in cell parameters are only used when they are defined by crystal symmetry. An approximate (isotropic) treatment of cell e.s.d.'s is used for estimating e.s.d.'s involving l.s. planes.
Refinement. Refinement of *F*^2^ against ALL reflections. The weighted *R*-factor *wR* and goodness of fit *S* are based on *F*^2^, conventional *R*-factors *R* are based on *F*, with *F* set to zero for negative *F*^2^. The threshold expression of *F*^2^ > σ(*F*^2^) is used only for calculating *R*-factors(gt) *etc*. and is not relevant to the choice of reflections for refinement. *R*-factors based on *F*^2^ are statistically about twice as large as those based on *F*, and *R*- factors based on ALL data will be even larger.

### Fractional atomic coordinates and isotropic or equivalent isotropic displacement parameters (Å^2^)

**Table:**

	*x*	*y*	*z*	*U*_iso_*/*U*_eq_	Occ. (<1)
O1	0.19392 (11)	0.0929 (2)	0.5063 (2)	0.0698 (7)	
N1	0.23113 (12)	0.2060 (3)	0.2915 (2)	0.0563 (7)	
H1N	0.2239	0.2356	0.1850	0.068*	
C1	0.28809 (14)	0.3034 (3)	0.3784 (3)	0.0502 (7)	
C2	0.34834 (14)	0.2312 (3)	0.4711 (3)	0.0519 (7)	
C3	0.40102 (15)	0.3326 (4)	0.5567 (3)	0.0618 (8)	
C4	0.39304 (17)	0.4983 (4)	0.5429 (3)	0.0733 (9)	
H4	0.4278	0.5652	0.6002	0.088*	
C5	0.33513 (19)	0.5685 (4)	0.4467 (4)	0.0789 (10)	
H5	0.3320	0.6809	0.4369	0.095*	
C6	0.28169 (17)	0.4699 (3)	0.3649 (3)	0.0652 (8)	
H6	0.2418	0.5157	0.3012	0.078*	
C7	0.18685 (15)	0.1087 (3)	0.3635 (3)	0.0533 (7)	
C8	0.12776 (15)	0.0097 (3)	0.2593 (3)	0.0636 (8)	
C9	0.1112 (4)	0.0650 (10)	0.0899 (5)	0.086 (2)	0.743 (14)
H9A	0.0865	0.1682	0.0853	0.128*	0.743 (14)
H9B	0.0801	−0.0136	0.0309	0.128*	0.743 (14)
H9C	0.1566	0.0752	0.0465	0.128*	0.743 (14)
C10	0.0569 (3)	0.0106 (12)	0.3340 (8)	0.100 (3)	0.743 (14)
H10A	0.0391	0.1201	0.3385	0.150*	0.743 (14)
H10B	0.0667	−0.0329	0.4380	0.150*	0.743 (14)
H10C	0.0202	−0.0548	0.2727	0.150*	0.743 (14)
C11	0.1582 (4)	−0.1659 (6)	0.2620 (10)	0.101 (3)	0.743 (14)
H11A	0.2051	−0.1661	0.2242	0.151*	0.743 (14)
H11B	0.1242	−0.2338	0.1964	0.151*	0.743 (14)
H11C	0.1640	−0.2066	0.3672	0.151*	0.743 (14)
C9A	0.1107 (14)	−0.144 (2)	0.347 (3)	0.091 (7)	0.153 (7)
H9D	0.0780	−0.1177	0.4215	0.136*	0.153 (7)
H9E	0.1558	−0.1881	0.4012	0.136*	0.153 (7)
H9F	0.0877	−0.2228	0.2742	0.136*	0.153 (7)
C10A	0.1509 (13)	−0.029 (3)	0.0996 (17)	0.082 (7)	0.153 (7)
H10D	0.1342	0.0566	0.0273	0.122*	0.153 (7)
H10E	0.1293	−0.1299	0.0618	0.122*	0.153 (7)
H10F	0.2038	−0.0366	0.1096	0.122*	0.153 (7)
C11A	0.0616 (9)	0.129 (2)	0.242 (3)	0.082 (6)	0.153 (7)
H11D	0.0791	0.2375	0.2295	0.124*	0.153 (7)
H11E	0.0374	0.1235	0.3336	0.124*	0.153 (7)
H11F	0.0274	0.0996	0.1517	0.124*	0.153 (7)
C9B	0.072 (2)	−0.059 (7)	0.360 (6)	0.085 (11)	0.104 (13)
H9G	0.0440	0.0291	0.3957	0.128*	0.104 (13)
H9H	0.0978	−0.1152	0.4480	0.128*	0.104 (13)
H9I	0.0391	−0.1324	0.2983	0.128*	0.104 (13)
C11B	0.089 (3)	0.128 (6)	0.138 (7)	0.111 (19)	0.104 (13)
H11G	0.0842	0.2324	0.1855	0.167*	0.104 (13)
H11H	0.0406	0.0873	0.0987	0.167*	0.104 (13)
H11I	0.1173	0.1392	0.0528	0.167*	0.104 (13)
C10B	0.173 (2)	−0.115 (6)	0.180 (7)	0.095 (11)	0.104 (13)
H10G	0.1773	−0.0790	0.0753	0.142*	0.104 (13)
H10H	0.1490	−0.2185	0.1756	0.142*	0.104 (13)
H10I	0.2216	−0.1242	0.2383	0.142*	0.104 (13)
C12	0.35791 (18)	0.0495 (4)	0.4751 (4)	0.0746 (9)	
H12A	0.3305	0.0027	0.3830	0.112*	
H12B	0.3401	0.0065	0.5662	0.112*	
H12C	0.4093	0.0234	0.4785	0.112*	
C13	0.46617 (16)	0.2627 (5)	0.6612 (4)	0.0859 (11)	
H13A	0.4976	0.2062	0.5987	0.129*	
H13B	0.4490	0.1883	0.7340	0.129*	
H13C	0.4935	0.3490	0.7175	0.129*	

### Atomic displacement parameters (Å^2^)

**Table:**

	*U*^11^	*U*^22^	*U*^33^	*U*^12^	*U*^13^	*U*^23^
O1	0.0848 (15)	0.0745 (15)	0.0504 (12)	−0.0125 (11)	0.0108 (9)	0.0006 (10)
N1	0.0721 (15)	0.0500 (14)	0.0461 (12)	−0.0083 (12)	0.0054 (10)	0.0016 (10)
C1	0.0617 (17)	0.0420 (16)	0.0468 (13)	−0.0051 (12)	0.0073 (12)	−0.0018 (12)
C2	0.0576 (16)	0.0471 (17)	0.0527 (14)	0.0025 (13)	0.0132 (12)	0.0018 (12)
C3	0.0570 (17)	0.071 (2)	0.0577 (16)	−0.0049 (15)	0.0082 (13)	0.0023 (15)
C4	0.074 (2)	0.069 (2)	0.073 (2)	−0.0191 (17)	−0.0008 (16)	−0.0073 (16)
C5	0.104 (3)	0.0400 (18)	0.089 (2)	−0.0114 (17)	0.001 (2)	−0.0069 (16)
C6	0.076 (2)	0.0459 (18)	0.0706 (18)	0.0028 (15)	−0.0011 (15)	0.0033 (14)
C7	0.0631 (17)	0.0461 (17)	0.0515 (16)	0.0017 (13)	0.0104 (12)	0.0003 (12)
C8	0.0728 (19)	0.0537 (18)	0.0629 (18)	−0.0116 (14)	0.0046 (14)	−0.0043 (14)
C9	0.085 (4)	0.100 (5)	0.064 (3)	−0.027 (3)	−0.013 (3)	−0.001 (3)
C10	0.081 (3)	0.122 (6)	0.098 (4)	−0.030 (4)	0.016 (3)	−0.010 (4)
C11	0.135 (5)	0.051 (3)	0.111 (5)	−0.012 (3)	−0.004 (4)	−0.017 (3)
C9A	0.095 (11)	0.080 (9)	0.101 (10)	−0.016 (8)	0.024 (8)	0.008 (8)
C10A	0.085 (10)	0.085 (11)	0.073 (8)	−0.005 (8)	0.006 (7)	−0.008 (8)
C11A	0.076 (9)	0.085 (9)	0.083 (10)	−0.004 (7)	0.000 (7)	−0.011 (8)
C9B	0.084 (13)	0.082 (15)	0.092 (13)	−0.006 (9)	0.018 (9)	0.011 (9)
C11B	0.11 (2)	0.12 (2)	0.11 (2)	−0.002 (10)	0.008 (10)	−0.005 (10)
C10B	0.103 (13)	0.092 (14)	0.092 (14)	−0.008 (9)	0.020 (9)	−0.013 (10)
C12	0.082 (2)	0.061 (2)	0.082 (2)	0.0128 (16)	0.0142 (17)	0.0059 (16)
C13	0.065 (2)	0.110 (3)	0.081 (2)	−0.001 (2)	0.0016 (16)	0.006 (2)

### Geometric parameters (Å, °)

**Table:**

O1—C7	1.229 (3)	C10—H10B	0.96
N1—C7	1.350 (3)	C10—H10C	0.96
N1—C1	1.440 (3)	C11—H11A	0.96
N1—H1N	0.94	C11—H11B	0.96
C1—C6	1.378 (4)	C11—H11C	0.96
C1—C2	1.401 (3)	C9A—H9D	0.96
C2—C3	1.405 (4)	C9A—H9E	0.96
C2—C12	1.505 (4)	C9A—H9F	0.96
C3—C4	1.374 (4)	C10A—H10D	0.96
C3—C13	1.505 (4)	C10A—H10E	0.96
C4—C5	1.378 (4)	C10A—H10F	0.96
C4—H4	0.93	C11A—H11D	0.96
C5—C6	1.386 (4)	C11A—H11E	0.96
C5—H5	0.93	C11A—H11F	0.96
C6—H6	0.93	C9B—H9G	0.96
C7—C8	1.539 (4)	C9B—H9H	0.96
C8—C9	1.522 (4)	C9B—H9I	0.96
C8—C10	1.525 (5)	C11B—H11G	0.96
C8—C9A	1.529 (8)	C11B—H11H	0.96
C8—C10A	1.530 (8)	C11B—H11I	0.96
C8—C9B	1.533 (8)	C10B—H10G	0.96
C8—C11B	1.534 (8)	C10B—H10H	0.96
C8—C10B	1.539 (8)	C10B—H10I	0.96
C8—C11	1.547 (5)	C12—H12A	0.96
C8—C11A	1.547 (8)	C12—H12B	0.96
C9—H9A	0.96	C12—H12C	0.96
C9—H9B	0.96	C13—H13A	0.96
C9—H9C	0.96	C13—H13B	0.96
C10—H10A	0.96	C13—H13C	0.96
			
C7—N1—C1	121.8 (2)	C8—C11—H11B	109.5
C7—N1—H1N	126.2	H11A—C11—H11B	109.5
C1—N1—H1N	111.0	C8—C11—H11C	109.5
C6—C1—C2	121.4 (2)	H11A—C11—H11C	109.5
C6—C1—N1	117.5 (2)	H11B—C11—H11C	109.5
C2—C1—N1	121.1 (2)	C8—C9A—H9D	109.5
C1—C2—C3	118.4 (2)	C8—C9A—H9E	109.5
C1—C2—C12	120.9 (2)	H9D—C9A—H9E	109.5
C3—C2—C12	120.6 (3)	C8—C9A—H9F	109.5
C4—C3—C2	119.1 (3)	H9D—C9A—H9F	109.5
C4—C3—C13	119.8 (3)	H9E—C9A—H9F	109.5
C2—C3—C13	121.1 (3)	C8—C10A—H10D	109.5
C3—C4—C5	122.1 (3)	C8—C10A—H10E	109.5
C3—C4—H4	119.0	H10D—C10A—H10E	109.5
C5—C4—H4	119.0	C8—C10A—H10F	109.5
C4—C5—C6	119.4 (3)	H10D—C10A—H10F	109.5
C4—C5—H5	120.3	H10E—C10A—H10F	109.5
C6—C5—H5	120.3	C8—C11A—H11D	109.5
C1—C6—C5	119.5 (3)	C8—C11A—H11E	109.5
C1—C6—H6	120.2	H11D—C11A—H11E	109.5
C5—C6—H6	120.2	C8—C11A—H11F	109.5
O1—C7—N1	122.6 (2)	H11D—C11A—H11F	109.5
O1—C7—C8	119.9 (2)	H11E—C11A—H11F	109.5
N1—C7—C8	117.5 (2)	C8—C9B—H9G	109.5
C9—C8—C10	109.7 (3)	C8—C9B—H9H	109.5
C9A—C8—C10A	112.2 (7)	H9G—C9B—H9H	109.5
C9B—C8—C11B	110 (3)	C8—C9B—H9I	109.5
C9B—C8—C10B	117 (3)	H9G—C9B—H9I	109.5
C11B—C8—C10B	110 (3)	H9H—C9B—H9I	109.5
C9—C8—C7	115.7 (3)	C8—C11B—H11G	109.5
C10—C8—C7	108.6 (3)	C8—C11B—H11H	109.5
C9A—C8—C7	108.8 (10)	H11G—C11B—H11H	109.5
C10A—C8—C7	112.1 (9)	C8—C11B—H11I	109.5
C9B—C8—C7	109 (2)	H11G—C11B—H11I	109.5
C11B—C8—C7	107 (2)	H11H—C11B—H11I	109.5
C10B—C8—C7	103.6 (16)	C8—C10B—H10G	109.5
C9—C8—C11	108.5 (3)	C8—C10B—H10H	109.5
C10—C8—C11	108.8 (3)	H10G—C10B—H10H	109.5
C7—C8—C11	105.3 (3)	C8—C10B—H10I	109.5
C9A—C8—C11A	111.3 (7)	H10G—C10B—H10I	109.5
C10A—C8—C11A	110.6 (7)	H10H—C10B—H10I	109.5
C8—C9—H9A	109.5	C2—C12—H12A	109.5
C8—C9—H9B	109.5	C2—C12—H12B	109.5
H9A—C9—H9B	109.5	H12A—C12—H12B	109.5
C8—C9—H9C	109.5	C2—C12—H12C	109.5
H9A—C9—H9C	109.5	H12A—C12—H12C	109.5
H9B—C9—H9C	109.5	H12B—C12—H12C	109.5
C8—C10—H10A	109.5	C3—C13—H13A	109.5
C8—C10—H10B	109.5	C3—C13—H13B	109.5
H10A—C10—H10B	109.5	H13A—C13—H13B	109.5
C8—C10—H10C	109.5	C3—C13—H13C	109.5
H10A—C10—H10C	109.5	H13A—C13—H13C	109.5
H10B—C10—H10C	109.5	H13B—C13—H13C	109.5
C8—C11—H11A	109.5		
			
C7—N1—C1—C6	−116.5 (3)	O1—C7—C8—C9	166.5 (5)
C7—N1—C1—C2	64.6 (3)	N1—C7—C8—C9	−16.0 (5)
C6—C1—C2—C3	2.9 (4)	O1—C7—C8—C10	42.6 (5)
N1—C1—C2—C3	−178.2 (2)	N1—C7—C8—C10	−139.9 (5)
C6—C1—C2—C12	−175.1 (2)	O1—C7—C8—C9A	−24.7 (11)
N1—C1—C2—C12	3.8 (4)	N1—C7—C8—C9A	152.8 (11)
C1—C2—C3—C4	−2.1 (4)	O1—C7—C8—C10A	−149.4 (11)
C12—C2—C3—C4	176.0 (3)	N1—C7—C8—C10A	28.1 (11)
C1—C2—C3—C13	178.7 (2)	O1—C7—C8—C9B	16 (2)
C12—C2—C3—C13	−3.3 (4)	N1—C7—C8—C9B	−166 (2)
C2—C3—C4—C5	−0.4 (5)	O1—C7—C8—C11B	135 (3)
C13—C3—C4—C5	178.9 (3)	N1—C7—C8—C11B	−48 (3)
C3—C4—C5—C6	2.1 (5)	O1—C7—C8—C10B	−109 (3)
C2—C1—C6—C5	−1.2 (4)	N1—C7—C8—C10B	69 (3)
N1—C1—C6—C5	179.8 (3)	O1—C7—C8—C11	−73.8 (5)
C4—C5—C6—C1	−1.3 (5)	N1—C7—C8—C11	103.7 (5)
C1—N1—C7—O1	−2.5 (4)	O1—C7—C8—C11A	92.6 (11)
C1—N1—C7—C8	−180.0 (2)	N1—C7—C8—C11A	−89.9 (11)

### Hydrogen-bond geometry (Å, °)

**Table:**

*D*—H···*A*	*D*—H	H···*A*	*D*···*A*	*D*—H···*A*
N1—H1N···O1^i^	0.94	2.11	2.966 (3)	151

Symmetry codes: (i) *x*, −*y*+1/2, *z*−1/2.
